# Factors Associated with the Chance of Carrying out a Primary Cesarean in a University Hospital

**DOI:** 10.1055/s-0042-1748976

**Published:** 2022-06-06

**Authors:** Maria Celeste Osório Wender, Rafaela Girardi Duarte, Gabriel Cardozo Muller, Rosaura Rolim Cavalheiro, Yanick Adolfo Leal Correia Silva, Cristiane Carboni, Edimárlei Gonsales Valério

**Affiliations:** 1 Postgraduate Program in Health Sciences: Gynecology and Obstetrics, Universidade Federal do Rio Grande do Sul (UFRGS), Porto Alegre, RS, Brazil; 2 Department of Gynecology and Obstetrics, Faculty of Medicine (FAMED), Universidade Federal do Rio Grande do Sul (UFRGS), Porto Alegre, RS, Brazil; 3 Faculty of Medicine (FAMED), Universidade Federal do Rio Grande do Sul (UFRGS), Porto Alegre, RS, Brazil; 4 Postgraduate Program in Epidemiology, Department of Social Medicine, Faculty of Medicine (FAMED), Universidade Federal do Rio Grande do Sul (UFRGS), Porto Alegre, RS, Brazil; 5 Service of Obstetrics and Gynecology, Hospital de Clínicas de Porto Alegre (HCPA), Porto Alegre, RS, Brazil

**Keywords:** cesarean section, indication, associated factors, cesariana, indicação, fatores associados

## Abstract

**Objective** The present study seeks to identify the associated factors that increased primary cesarean delivery rates.

**Methods** This was a cross-sectional study that evaluated the number of primary cesarean sections performed in the years 2006 and 2018 at the Hospital de Clínicas de Porto Alegre (HCPA, in the Portuguese acronym), through the collection of data from the medical records of the patients.

**Results** Advanced maternal age, twin pregnancy, and higher body mass index (BMI) became more frequent in 2018 in comparison with 2006. To mitigate the impact of confounding in comparisons among groups, we made an adjustment by propensity scores and detected significant differences when comparing both age groups on twin pregnancy rates, gestational diabetes mellitus, and thyroid disease.

**Conclusion** Data from the present study can be used to prevent and improve the management of morbidities, impacting on better outcomes in obstetrical practice.

## Resumo

**Objetivo** O presente estudo busca identificar os fatores associados que aumentam as taxas de partos cesáreos primários.

**Métodos** Estudo transversal, avaliando o número de cesáreas primárias realizadas nos anos de 2006 e 2018 no Hospital de Clínicas de Porto Alegre (HCPA), por meio da coleta de dados nos prontuários das pacientes.

**Resultados** Idade materna avançada, gravidez gemelar e índice de massa corporal (IMC) mais elevado tornaram-se mais frequentes em 2018. Para mitigar o impacto dos fatores de confusão nas comparações entre os grupos, fizemos um ajuste por escores de propensão e detectamos diferenças significativas nas taxas de gravidez gemelar, diabetes mellitus gestacional e doença da tireoide.

**Conclusão** Os dados do presente estudo podem ser utilizados para prevenir e melhorar o manejo de morbidades, impactando em melhores resultados na prática obstétrica.

## Introduction

In recent years, the rapid increase in cesarean section rates without clear evidence of concomitant decrease in maternal or neonatal morbidity or mortality raises significant concern that cesarean delivery is overused. 1

The global increase in the number of cesarean sections is accompanied by an increase in maternal and neonatal morbidity and mortality. Some of the outcomes observed are a higher risk of complications in childbirth, from an anesthetic point of view, since analgesia is performed at the surgical level. As a surgical procedure, cesarean section offers a higher risk of complications when compared with vaginal delivery, as well as longer hospitalization and postpartum recovery time. Other complications related to the surgical event include increased risk of surgical wound hematoma, cardiac events, puerperal infection, and long-term complications of abdominal surgery such as adhesions and incisional hernia formation. 1 2 3

It is worth noting that an increased number of cesarean sections may also be related to a higher risk of placenta accreta, intestinal injury, ureteral injury, need for postoperative ventilation, hospitalization in the intensive care unit, hysterectomy, and blood transfusion. In addition, most uterine ruptures are associated with an attempt to labor after a previous cesarean section. 2 3

Despite attempts to reduce the risk of these adverse outcomes, the current high rates of cesarean deliveries are not accompanied by a reduction in maternal and neonatal morbidity and mortality, forgetting the art and experience of vaginal delivery is a serious challenge to the nature and training of future obstetricians. In an overview, the journey of childbirth care from the 21 ^st^ century onwards ends in increasing rates of cesarean sections around the world. 1 2 3

Primary cesarean is considered to be performed in women who have never undergone this procedure before. As our objective was to determine associated factors with primary cesarean sections to provide data on future strategies to potentially reduce elective/nonessential indications, we collected data corresponding to age, ethnicity, residence, body mass index (BMI), gestational age at birth, number of fetuses, fetal presentation, cesarean indications, and maternal comorbidities. In addition, since our hospital is a regional reference center for high-risk pregnancies, we collected the city of residence of the patients. The indications for cesarean sections were also analyzed, comparing the two years observed in the present study in relation to the conditions mentioned below in the methods section.

## Methods

Our group designed a cross-sectional study with data (collected from medical records) of all patients who underwent primary cesarean at a university hospital in southern Brazil (Hospital de Clínicas de Porto Alegre [HCPA, in the Portuguese acronym, Porto Alegre, RS, Brazil) in the years 2006 and 2018. The years 2006 and 2018 were chosen because our database was already filled with information from these years, and because we also had the objective of comparing the factors associated with the chance of primary cesarean section in different years.

All patients who underwent primary cesarean at the HCPA in 2006 and 2018 were included in the present study. Data were collected from the collection of data from the medical records of these patients.

The indications for cesarean sections were analyzed, comparing the two years regarding the following conditions: whether the indication was elective or urgent, noncephalic presentations, multiple pregnancies, nonreassuring fetal condition, cephalopelvic disproportion, failure in induction of labor, antepartum hemorrhage, placental abruption, placenta previa, HIV-positive, fetal malformations, active herpes, and macrosomia.

Our project was approved by the Research Ethics Committee of the HCPA (Letter of Approval number 2020/0672) and was also forwarded to and approved by Plataforma Brasil for publication (CAAE 40587620.7.0000.5327).

All analyses were made using R v4.0.1 (R Foundation for Statistical Computing, Vienna, Austria) and Rstudio v1.4.1717 (RStudio Team. RStudio: Integrated Development Environment for R).

To analyze differences between both groups, the Fisher exact test or the chi-squared test were used for qualitative variables and, for quantitative ones, the Mann-Whitney test for independent samples (as the majority presented an asymmetrical distribution) was used. For comparisons between proportions, the prevalence ratios (PRs) accompanied by their confidence intervals (CIs) at 95% were expressed, with differences whose CI did not contain the unit and whose two-sided p-value was below the 5% significance level were considered significant.

To adjust for potential confounders, we used a propensity score matching through a logistic regression model with covariates defined by theory and by those baseline characteristics that presented statistical significance in comparison among groups. To prevent violation of logistic regression assumptions, we categorized continuous covariates (BMI, gestational week, and age).

## Results

In 2006, there were a total of 3,919 births, 2,636 vaginal births, and 1,239 cesarean sections. Among the total number of cesarean sections, the number of primary cesarean sections was 771, composing one of the analysis groups of our study ( Table 1 ).

**Table 1:** Births in 2006 and 2018

Year	Vaginal births	Cesarean sections	Primary Cesareans	Births	Number of pregnant women
2006	2.636	1.239	771	3.919	3.870
2018	2.181	1.334	722	3.567	3.507

In 2018, there were 3,567 births, with 2,181 of them by vaginal delivery and 1,334 by cesarean sections. The number of primary cesarean sections in 2018 was 722, making up the other analysis group in our study. Pregnant women who underwent cesarean sections were older and had more comorbidities in 2018 compared with 2006. Pregnant women grouped in 2018 also had a higher BMI. These variables (age, morbidity presence, BMI), along with blood type, gemelarity, and gestational week ( Table 2 ) were used for propensity scores matching to adjust to these potential confounders. After this matching, our sample comprised 862 patients divided equally into both groups. Then, the epidemiological profile of pregnant women in these 2 years were compared. We considered morbidity as the presence of common or uncommon diseases during pregnancy, such as gestational diabetes mellitus (GDM) and pregestational diabetes mellitus, systemic arterial hypertension, pre-eclampsia and eclampsia, cardiopathy, nephropathy, hepatopathy, and thyroid disease.

**Table 2:** Baseline factors – frequencies by year

	Year		*p-value*
	2006 (771)	2018 (722)	
Age (years old)	23.00 (19.00–29.00)	26.00 (22.00–32.00)	< 0.001
Age, categorical (years old) (%)			
< 18	96 (12.5)	28 (3.9)	< 0.001
18–34	600 (77.8)	566 (78.4)	
35–39	55 (7.1)	94 (13.0)	
≥ 40	20 (2.6)	34 (4.7)	
BMI (kg/m ^2^ )	29.20 (26.30–33.50)	31.60 (28.22–36.10)	< 0.001
BMI, categorical (%)			
Eutrophic	93 (13.7)	44 (9.1)	< 0.001
Overweight	283 (41.6)	137 (28.4)	
Obesity I	173 (25.4)	148 (30.7)	
Obesity II or III	132 (19.4)	153 (31.7)	
Gestational age at birth (weeks)	39.00 (37.00–40.00)	39.00 (37.00–40.00)	0.05
Gestational age, categorical			
Extremely premature (< 28 weeks)	23 (3.0)	13 (1.8)	0.327
Premature (28–36 weeks)	158 (20.5)	152 (21.1)	
Term (≥ 37 weeks)	590 (76.5)	557 (77.1)	
Number of fetuses (%)			
Twin	26 (3.4)	42 (5.8)	0.03
Triplet	3 (0.4)	3 (0.4)	1.00
Blood type (%)			
A	316 (42.0)	255 (35.3)	0.057
AB	31 (4.1)	28 (3.9)	
B	82 (10.9)	83 (11.5)	
O	324 (43.0)	356 (49.3)	
Morbidity (%)	267 (34.6)	357 (49.4)	< 0.001

Abbreviation: BMI, body mass index.

Values are expressed either in absolute value and percentage or in median and interquartile range.

When comparing both groups, there was a significant difference between the 2 years in twin pregnancy rates, GDM, and thyroid disease even after the adjusted analysis ( Table 3 ). It is important to emphasize that it was not possible to obtain the BMI data of 329 patients (22%) because the deliveries occurred at the time of admission, by emergency cesarean. In contrast, after the adjusted analysis, there was no significance in the two years for HIV-positive pregnant women and depression.

**Table 3:** Comparison of morbimorbidities matched by propensity scores

Year					
	2006 (431)	2018 (431)	PR	95%CI	*p-value*
Ethnicity, non-white (%)	87 (20.2)	102 (23.7)	1.10	0.95–1.29	0.20
Porto Alegre (%)	276 (64.0)	288 (66.8)	1.06	0.92–1.23	0.39
Systemic arterial hypertension (%)	45 (10.4)	44 (10.2)	0.99	0.79–1.23	0.91
Severe pre-eclampsia (%)	20 (4.6)	13 (3.0)	0.78	0,51–1.20	0.26
Mild pre-eclampsia (%)	29 (6.7)	24 (5.6)	0.90	0.66–1.22	0.50
Rh factor, + (%)	387 (89.8)	395 (91.6)	1.12	0,87–1.44	0.30
Eclampsia (%)	4 (0.9)	1 (0.2)	0,55	0.24–1.27	0.16
Musculoskeletal diseases (%)	6 (1.4)	7 (1.6)	1.08	0.65–1.79	0.77
HIV positive (%)	12 (2.8)	14 (3.2)	1.08	0.75–1.55	0.68
Hepatopathy (%)	10 (2.3)	7 (1.6)	0.82	0.46–1.45	0,50
Cardiopathy (%)	4 (0.9)	8 (1.9)	1.34	0.89–2.01	0.16
Thyroid diseases (%)	2 (0.5)	16 (3.7)	1.81	1.51–2.16	< 0.0001
Nephropathy (%)	15 (3.5)	17 (3.9)	1.07	0.76–1.49	0,71
Pregestational diabetes mellitus (%)	7 (1.6)	10 (2.3)	1.18	0.79–1.77	0.42
Gestational diabetes mellitus (%)	17 (3.9)	39 (9.0)	1.43	1.19–1.73	< 0.0001
Depression (%)	7 (1.6)	14 (3.2)	1.34	0.99–1.83	0.06

Abbreviations: CI, confidence interval; PR, prevalence ratio.

Frequencies are aseparated by year. Values are expressed either by absolute value and percentage or by median and interquartile range. ⧪viral load > 1,000 copies/ml or unknown.

There was no statistically significant difference in the percentage of indications for elective and nonelective primary cesarean sections between the 2 years ( *p* = 0.2; PR: 1.10; 95%CI: 0.93–1.30), even in the unadjusted analysis. Most of the variables analyzed did not show statistically significant differences in the indications of primary cesarean sections. However, there were significant differences in crude analysis comparing the two groups regarding premature rupture of membranes, fetal malformations, and antepartum hemorrhage. In these comparisons, there was also significance after matching by propensity scores ( Table 4 ).

**Table 4:** Indications for primary cesareans–frequencies by year

Year					
	2006 (431)	2018 (431)	PR	95%CI	*p-value*
Indication					
Elective cesarean	113 (26.2)	98 (22.7)	1	−	0.25
Nonelective cesarean	318 (73.8)	333 (78.3)	1.10	0.93–1.30	
Noncephalic presentation	62 (14.4)	70 (16.2)	1.07	0.89–1.28	0.43
Multiple pregnancy (twin + triplet)	1 (0.2)	1 (0.2)	1	0.24–4.00	1
Nonreassuring fetal condition	110 (25.5)	119 (27.6)	1,05	0.91–1.22	0.48
Cephalopelvic disproportion	157 (36.4)	154 (35.7)	0,98	0.86–1.13	0.83
Induction labor failure &	36 (8.4)	49 (11.4)	1,17	0.96–1.43	0.11
Premature rupture of membranes	77 (17.9)	103 (23.9)	1.19	1.02–1.38	0.02
Antepartum hemorrhage	8 (1.9)	16 (3.7)	1,35	1.01–1.80	0.05
Placental abruption	10 (2.3)	11 (2.6)	1.05	0.69–1.59	0.82
Placenta previa	2 (0.5)	2 (0.5)	1,00	0.37–2.67	1.00
Fetal malformations	6 (1.4)	20 (4.6)	1,56	1.25–1.95	0,00
Active herpes	4 (0.9)	6 (1.4)	1,20	0.72–2.00	0,48
Macrosomia	42 (9.7)	44 (10.2)	1,03	0.82–1.28	0,82

Abbreviations: CI, confidence interval; PR, prevalence ratio.

Frequencies are separated by year. Values are expressed either by absolute value and percentage or by median and interquartile range.

& with indication of interruption without labor or any obstetric indication that prevents delivery.

## Discussion

The World Health Organization (WHO) advocates that every effort should be made to provide cesarean sections to women in need, rather than achieving a specific goal. For low-risk conditions, cesarean delivery seems to pose more maternal risk than vaginal delivery. Although the indications for cesarean deliveries are established, the choice for cesarean deliveries has increased globally both in low-, middle- and high-income countries. This trend, however, was not accompanied by significant maternal and perinatal benefits. On the contrary, the increase in cesarean delivery rates was not associated with any demonstrable improvement in maternal or neonatal morbidity or mortality. 3 4 5 6

Advanced maternal age, defined as pregnancy in women > 35 years old, is associated with a potential clinical risk of complications such as fetal growth restriction, pre-eclampsia, placental abruption, preterm delivery, and stillbirth. In addition, systematic reviews and meta-analyses have shown the association between advanced maternal age and increased risk of cesarean delivery. 7 8 Our study also showed that advanced maternal age was associated with a greater number of primary cesarean sections in 2018 compared with 2006.

Gestational diabetes mellitus confers an increased risk of serious complications during pregnancy both for the mother and the child, including cesarean delivery, shoulder dystocia, macrosomia, and neonatal hypoglycemia. 9 In other studies, pregnant women with GDM had overall cesarean rates of 35.3%. Simultaneously, compared with nondiabetic pregnant women, the reported cesarean rate was 1.52 times higher in patients with GDM. 10 11 In this sense, our findings are similar to what is found in the literature regarding the greater number of pregnancies that evolved to cesarean delivery in women with GDM. Even so, this increase may be partially explained by changes in diagnostic criteria between the two years. 12

Premature rupture of membranes, despite not being among the indications for primary cesarean sections, showed a statistically significant increase when comparing the years 2006 and 2018. We relate this fact to the possibility that other variables are associated with the condition, such as nonreassuring fetal condition and induction failure, among others that configure indication for primary cesarean.

Twin pregnancies were also more frequent in 2018 in the unadjusted analysis, and, therefore, were used for matching. However, when added to the triplet pregnancies, there was no statistically significant difference in the indications for cesarean sections between the two years evaluated. In vitro fertilization (IVF) is a safe and highly effective treatment for infertility. 13 14 However, risks of obstetric and perinatal morbidity, such as hypertensive disorders of pregnancy, GDM, cesarean section, placenta accreta, premature delivery, and low birthweight, have been associated with IVF. These adverse outcomes are largely due to an increased risk of multiple pregnancies in IVF, as several perinatal complications increase with multiple pregnancies, including fetal anomalies, pre-eclampsia, and GDM. 13 14 We think that the increase in the number of twin pregnancies in 2018 is due, in part, to the increase in assisted reproduction techniques.

According to Hannah et al., 15 there is a consensus that planned cesarean delivery is better than planned vaginal delivery for the delivery of the fetus at breech presentation, if the fetus is compromised, if the fetus is large, or if it has a congenital anomaly that can cause a mechanical problem in vaginal delivery. Also, these authors concluded that a planned cesarean policy is substantially better for the single fetus at term breech presentation, with the benefits being greatest in countries reporting lower perinatal mortality rates. 15 A planned cesarean policy is not associated with an increased risk of serious problems for the mother in the 1 ^st^ 6 weeks after delivery. However, some results show that a subsequent delivery after a pelvic cesarean delivery is associated with an increase in maternal and child morbidity, regardless of the type of the subsequent delivery. 15 16

In recent years, in addition to the increase in the number of cesarean sections, there is a trend toward an increase in the number of elective cesarean sections, many of them occurring at the request of the mother. Despite this increase, some studies suggest that maternal and neonatal morbidity and mortality would not be reduced. 17 18 Our hospital serves predominantly patients from the public health system following strict guidelines and criteria for performing elective cesarean sections. This can justify the findings of our study, which presented no difference between elective and nonelective cesarean sections between 2006 and 2018.

In our study, there was no difference regarding abnormal fetal presentation as an indication for cesarean sections in the two years evaluated.

Before matching, there was a difference in comorbidity presence between the years of 2006 and 2018. After matching and including this variable, only thyroid disease was more frequent in 2018 than in 2006. Thyroid disease may be related to the increasing age of pregnant women, since there is an increase in the incidence of this disease with age, among other factors.

In addition to the limitations intrinsic to a cross-sectional study, we can add the loss of 22% of the given BMI, the fact that we were analyzing data from a tertiary hospital, and a sample that was probably biased. As external validity, we considered tertiary centers and centers in developing countries.

## Conclusion

Known risk factors for adverse events during pregnancy have become more frequent, including advanced maternal age, twin pregnancy, higher BMI, GDM, thyroid disease, and premature rupture of membranes. The present work provides data to reinforce institutional strategies for the prevention and proper management of morbidities in our hospital; therefore, it could reduce complications during pregnancy, as well as indications for primary cesarean sections.

## References

[1] Mahadik K Rising cesarean rates: are primary sections overused? J Obstet Gynaecol India 2019 69 06 483 489 10.1007/s13224-019-01246-y 31844361 PMC6889110

[2] Term Breech Trial 3-Month Follow-up Collaborative Group Hannah M E Hannah W J Hodnett E D Chalmers B Kung R Willan A Outcomes at 3 months after planned cesarean vs planned vaginal delivery for breech presentation at term: the international randomized Term Breech Trial JAMA 2002 287 14 1822 1831 10.1001/jama.287.14.1822 11939868

[3] National Institute of Child Health and Human Development Maternal-Fetal Medicine Units Network Silver R M Landon M B Rouse D J Leveno K J Spong C Y Thom E A Maternal morbidity associated with multiple repeat cesarean deliveries Obstet Gynecol 2006 107 06 1226 1232 16738145 10.1097/01.AOG.0000219750.79480.84

[4] Kingdon C Downe S Betran A P Non-clinical interventions to reduce unnecessary caesarean section targeted at organisations, facilities and systems: Systematic review of qualitative studies PLoS One 2018 13 09 e0203274 10.1371/journal.pone.0203274 30180198 PMC6122831

[5] Clark S L Belfort M A Dildy G A Herbst M A Meyers J A Hankins G D Maternal death in the 21st century: causes, prevention, and relationship to cesarean delivery Am J Obstet Gynecol 2008 199 01 360 3.6E6, discussion 91–92, e7–e11. 10.1016/j.ajog.2008.03.007 18455140

[6] Queenan J T Spong C Y Lockwood C J Protocols for high-risk pregnancies: an evidence-based approach7th ed.Hoboken: John Wiley & Sons; 2021

[7] Lean S C Derricott H Jones R L Heazell A EP Advanced maternal age and adverse pregnancy outcomes: A systematic review and meta-analysis PLoS One 2017 12 10 e0186287 10.1371/journal.pone.0186287 29040334 PMC5645107

[8] Bayrampour H Heaman M Duncan K A Tough S Comparison of perception of pregnancy risk of nulliparous women of advanced maternal age and younger age J Midwifery Womens Health 2012 57 05 445 453 10.1111/j.1542-2011.2012.00188.x 22954075

[9] Chiefari E Arcidiacono B Foti D Brunetti A Gestational diabetes mellitus: an updated overview J Endocrinol Invest 2017 40 09 899 909 10.1007/s40618-016-0607-5 28283913

[10] Wang J Chen K Jin X Li X An P Yang N Prognostic factors for cesarean section outcome of pregnant women with gestational diabetes mellitus: a systematic review and meta-analysis Diabetes Metab Syndr Obes 2019 12 913 929 10.2147/DMSO.S188293 31296993 PMC6596347

[11] Gorgal R Gonçalves E Barros M Namora G Magalhães A Rodrigues T Gestational diabetes mellitus: a risk factor for non-elective cesarean section J Obstet Gynaecol Res 2012 38 01 154 159 10.1111/j.1447-0756.2011.01659.x 21995455

[12] Paulo M S Abdo N M Bettencourt-Silva R Al-Rifai R H Gestational diabetes mellitus in Europe: a systematic review and meta-analysis of prevalence studies Front Endocrinol (Lausanne) 2021 12 691033 10.3389/fendo.2021.691033 34956073 PMC8698118

[13] Sullivan-Pyke C S Senapati S Mainigi M A Barnhart K T In Vitro fertilization and adverse obstetric and perinatal outcomes Semin Perinatol 2017 41 06 345 353 10.1053/j.semperi.2017.07.001 28818301 PMC5951714

[14] Foggin H Hutcheon J A Liauw J Making sense of harms and benefits: Assessing the numeric presentation of risk information in ACOG obstetrical clinical practice guidelines Patient Educ Couns 2021 S0738 3991(21)00576-0 10.1016/j.pec.2021.08.030 34509341

[15] Hannah M E Hannah W J Hewson S A Hodnett E D Saigal S Willan A R Planned caesarean section versus planned vaginal birth for breech presentation at term: a randomised multicentre trial Obstet Gynecol Surv 2001 56 03 132 134 10.1097/00006254-200103000-00007 11052579

[16] Macharey G Gissler M Rahkonen L Ulander V-M Väisänen-Tommiska M Nuutila M Breech presentation at term and associated obstetric risks factors-a nationwide population based cohort study Arch Gynecol Obstet 2017 295 04 833 838 10.1007/s00404-016-4283-7 28176014

[17] Belizán J M Althabe F Cafferata M L Health consequences of the increasing caesarean section rates Epidemiology 2007 18 04 485 486 10.1097/EDE.0b013e318068646a 17568221

[18] World Health Organization 2005 Global Survey on Maternal and Perinatal Health Research Group Villar J Carroli G Zavaleta N Donner A Wojdyla D Faundes A Maternal and neonatal individual risks and benefits associated with caesarean delivery: multicentre prospective study BMJ 2007 335(7628):1025 10.1136/bmj.39363.706956.55 17977819 PMC2078636

